# Alpha‐Synuclein as a Potential Biomarker for Inclusion Body Myositis in Blood and Muscle

**DOI:** 10.1111/nan.70019

**Published:** 2025-05-19

**Authors:** Tobias Mayer, Leila Scholle, Laura Foerster, Ilka Schneider, Gisela Stoltenburg‐Didinger, Karl‐Stefan Delank, Thomas Kendzierski, Anna Koelsch, Kathleen Kleeberg, Torsten Kraya, Lorenzo Barba, Steffen Naegel, Anne Schänzer, Markus Otto, Alexander Mensch

**Affiliations:** ^1^ Department of Neurology University Medicine Halle Halle (Saale) Germany; ^2^ Department of Neurology St. Georg Hospital Leipzig Leipzig Germany; ^3^ Institute of Cell and Neurobiology, Charité University Medicine Berlin Berlin Germany; ^4^ Department of Orthopedics, Trauma and Reconstructive Surgery University Medicine Halle Halle (Saale) Germany; ^5^ Department of Neurology Alfried Krupp Krankenhaus Rüttenscheid Essen Germany; ^6^ Institute of Neuropathology Justus‐Liebig University Giessen Germany; ^7^ Translational Neuroscience Network Giessen (TNNG) Justus Liebig University Giessen Giessen Germany

**Keywords:** biomarker, IBM, inclusion body, myositis, α‐synuclein

## Abstract

**Aims:**

Diagnosis of inclusion body myositis (IBM) is difficult and currently based on a combination of clinical and (immuno)histological findings. Biomarkers facilitating the diagnostic process are needed. Alpha‐synuclein (αSN) aggregates are a known histological feature of IBM, but there is a lack of information on their diagnostic relevance. Furthermore, serum αSN concentrations in IBM have not been investigated.

**Methods:**

Immunohistochemical staining for αSN was performed on 63 biopsies (19 IBM, 21 other inflammatory myopathies, 20 other myopathies and 3 healthy controls), and αSN reactive fibres were quantified. The serum concentration of αSN was determined by ELISA in 156 serum samples (11 IBM, 25 other inflammatory myopathies, 53 hereditary myopathies, 30 mitochondriopathies and 37 healthy controls).

**Results:**

The proportion of fibres with αSN immunoreactivity was significantly higher in IBM compared to all groups (*p* < 0.001) and discriminated IBM against all other neuromuscular disorders with a sensitivity of 79% and a specificity of 85%, which further improved when only non‐regenerating fibres were examined. In serum, αSN concentrations in IBM were generally not different from healthy controls. However, serum concentrations were inversely correlated with disease duration (*r* = −0.62, *p* = 0.04) and positively correlated with the IBM functional rating scale (*r* = 0.74, *p* = 0.01). Consequently, stratification according to these clinical parameters showed significantly lower serum αSN concentrations in late‐stage, more severely affected patients.

**Conclusions:**

αSN reactivity may serve as an additional immunohistochemical marker for IBM diagnosis. Furthermore, this study indicates that αSN serum concentrations decrease with disease duration and clinical deterioration. Therefore, serum αSN may be provisionally considered a monitoring biomarker in IBM, pending further studies.

## Graphical abstract

We examined alpha‐synuclein in muscle and serum samples of patients with IBM and other neuromuscular disorders. With the use of immunohistochemistry, we identify alpha‐synuclein muscular reactivity as a complementary diagnostic marker. Serum alpha‐synuclein levels measured by ELISA were not generally altered in IBM but correlated with disease duration and clinical severity, suggesting a potential role for serum alpha‐synuclein as a monitoring biomarker.

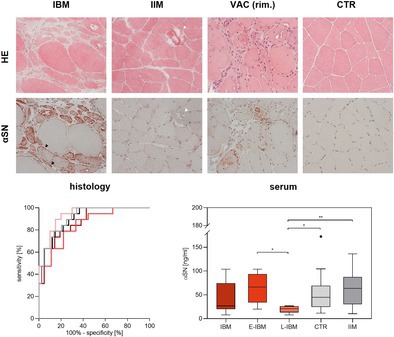

**Summary**

- Muscle fibres with alpha‐synuclein‐immunoreactive inclusions are more frequent in inclusion body myositis (IBM) than in non–IBM inflammatory myopathies, other myopathies and healthy controls.
- Alpha‐synuclein immunohistochemical staining of muscle biopsies has sufficient sensitivity (79%) and specificity (81%) to differentiate IBM from other inflammatory myopathies.
- Regenerating muscle fibres account for a proportion of alpha‐synuclein‐reactive muscle fibres, the exclusion of which results in higher sensitivity (84%) and specificity (86%) for distinguishing IBM from other inflammatory myopathies.
- Serum alpha‐synuclein concentrations are generally not altered in IBM but correlate with disease duration and clinical severity.
- Serum alpha‐synuclein may qualify as a monitoring biomarker in IBM.

## 1 Introduction

Inclusion body myositis (IBM) is one of the most common inflammatory myopathies in patients over the age of 50 years [1]. Initial symptoms typically include weakness of the quadriceps femoris muscle and the deep finger flexors, resulting in early impairment of ambulation and fine motor tasks [2, 3]. Other frequent features include weakness of foot extension, camptocormia and dysphagia [4, 5, 6, 7, 8, 9].

Both autoimmune and degenerative processes are involved in the pathogenesis of IBM [3]. Autoimmune features include autoantibodies against the cytosolic 5′‐nucleotidase 1A (cN1A) as well as oligoclonal expansion of highly differentiated cytotoxic T cells, release of proinflammatory cytokines such as IFNγ and upregulation of major histocompatibility complex (MHC) Classes I and II proteins [10, 11, 12, 13, 14, 15, 16, 17, 18, 19, 20]. However, most patients with IBM receive limited benefit from immunosuppressive treatment, which is why concomitant muscle degeneration has been considered to be an additional pathogenic mechanism [21, 22, 23]. Indeed, similar to neurodegenerative diseases, accumulation of proteins such as ubiquitin, (phosphorylated) tau protein, transactive response DNA–binding protein 43 kDa (TDP‐43) and p62 has been identified in muscle biopsies of IBM patients, especially in advanced stages of the disease [24, 25, 26, 27, 28]. Other degenerative features have also been reported, including impaired autophagy, mitochondrial abnormalities and impairment of the 26S‐ubiquitin‐proteasome system [29].

Despite the clinical and histological peculiarities of IBM, differentiation from other idiopathic inflammatory myopathies (IIM) and neuromuscular disorders (e.g., amyotrophic lateral sclerosis) remains challenging, and patients are frequently misdiagnosed [2, 30, 31]. This is accounted for partly by the slowly progressive nature of the disease and the lack of definite paraclinical characteristics. Therefore, several studies have focused on the diagnostic utility of particular histological features in IBM. To date, intramuscular aggregates of TDP‐43 and p62 proteins are considered the most accurate degenerative markers, along with other useful histological features such as COX‐/SDH + myofibres and MHC Classes I and II upregulation [20, 27, 32, 33, 34, 35]. However, these aggregates are more common in biopsies that also show other histological features of advanced disease (e.g., rimmed vacuoles [RV]) [32]. Hence, the value of immunohistochemical protein analysis may be limited in the early stages of the disease [36]. Initial studies reported a high specificity (85%–100%) of anti–cN1A antibody positivity, while more recent publications have not been able to replicate these findings [5, 10, 11, 37, 38, 39]. Furthermore, the sensitivity of anti‐cN1A in detecting IBM varies widely between studies, ranging from 36% to 70% [5, 10, 11, 37, 38]. Therefore, there is an urgent need for additional biomarkers to support the correct diagnosis and to monitor disease progression.

α‐Synuclein (αSN) is a presynaptic protein that is primarily recognised as the main constituent of Lewy bodies in Parkinson's disease (PD) and dementia with Lewy bodies (DLB) [40, 41, 42, 43]. αSN immunoreactivity has also been reported in muscle biopsies from IBM patients as punctate sarcoplasmic inclusions in the postsynaptic domain of neuromuscular junctions and in regenerating and necrotic muscle fibres [44]. To date, there are conflicting results regarding the expression and localisation of αSN deposits in IBM, and only one study has semiquantitatively assessed the expression of αSN in muscle homogenate samples of seven IBM cases, which was found to be 6.3 times higher than in control samples [7, 32, 45]. Moreover, serum concentrations of αSN, which have been investigated in PD patients with conflicting results, remain unexplored in patients with IBM [42].

Considering these issues, this pilot study aimed to investigate the diagnostic value of αSN immunoreactivity and concentration in muscle biopsies in a well‐characterised cohort of patients with IBM. Furthermore, we evaluated serum αSN concentrations in IBM and other muscle diseases and their associations with clinical and immunohistochemical data.

## 2 Materials and Methods

### 2.1 Patients and Samples

We aimed to compare the expression levels of αSN in muscle tissue and serum of IBM patients with those of other neuromuscular disorders and healthy controls. The study design allowed for prospective sampling and retrospective inclusion of biopsy specimens previously obtained during routine diagnostic procedures, with the patient's informed consent. This resulted in a different cohort size depending on the biomaterial used, with detailed information provided in Table 1.

**TABLE 1:** Epidemiological and paraclinical data of included patients.

	(Immuno‐)histochemical staining							
	*n*	Sex		Age (years)	Modified MRC‐sum score	Disease duration (years)	CK (μkat/L)	IBMFRS
		M	F					
IBM	19	12	7	65.68 ± 9.59	57.40 ± 9.00 (*n* = 15)	3.65 ± 3.30 (*n* = 17)	10.43 ± 5.34 (*n* = 18)	/
IIM	21	9	12	60.62 ± 10.06	60.11 ± 6.24 (*n* = 19)	3.30 ± 5.74 (*n* = 20)	61.96 ± 56.05 (*n* = 20)	/
IMNM	9	6	3	62.56 ± 8.88	56.44 ± 4.98	2.44 ± 6.60	93.79 ± 53.61	/
OLM	6	2	4	57.50 ± 15.81	62.60 ± 4.78 (*n* = 5)	2.83 ± 4.07	46.07 ± 58.30	/
PM‐M	4	0	4	62.75 ± 1.50	65.00 ± 7.39	6.50 ± 6.86	16.17 ± 14.35	/
DM	2	1	1	57.00 ± 1.41	61 (*n* = 1)	1 (*n* = 1)	53.97 (*n* = 1)	/
OM	20	9	11	54.70 ± 15.34	63.58 ± 5.38 (*n* = 12)	4.33 ± 3.04 (*n* = 9)	11.00 ± 10.28 (*n* = 13)	/
VAC (rim.)	9	6	3	58.56 ± 14.99	64.60 ± 3.44 (*n* = 5)	5.20 ± 3.42 (*n* = 5)	8.12 ± 2.58 (*n* = 5)	/
LDB3	3	2	1	68.67 ± 12.74	64.33 ± 3.79	5.67 ± 4.04	9.01 ± 2.96	/
FLNC	1	0	1	46	/	/	/	/
TIA1/SQSTM1	1	0	1	54	68	7	5.53	/
SQSTM1	1	1	0	53	62	2	8.05	/
MPD	1	1	0	33	/	/	/	/
NEUR	1	1	0	76	/	/	/	/
MFM	1	1	0	59	/	/	/	/
VAC (non‐rim.)	8	3	5	53.75 ± 17.49	60.80 ± 6.72 (*n* = 5)	4.50 ± 2.12 (*n* = 2)	16.96 ± 15.08 (*n* = 5)	/
GSD2	3	2	1	48.00 ± 26.00	62.00 ± 8.49 (*n* = 2)	4.50 ± 2.12 (*n* = 2)	5.69 ± 4.32 (*n* = 2)	/
GSD5	3	0	3	61.67 ± 14.50	60.00 ± 7.21	/	24.48 ± 15.28	/
CAM	1	0	1	45	/	/	/	/
SLONM	1	1	0	56	/	/	/	/
Non‐VAC	3	0	3	45.67 ± 8.51	68.00 ± 2.83 (*n* = 2)	2.00 ± 2.83 (*n* = 2)	5.84 ± 4.10 (*n* = 3)	/
DM2	3	0	3	45.67 ± 8.51	68.00 ± 2.83 (*n* = 2)	2.00 ± 2.83 (*n* = 2)	5.84 ± 4.10 (*n* = 3)	/
CTR	3	3	0	50.00 ± 12.17	67.67 ± 4.04	/	2.94 ± 1.64	/
Total	63	33	30	59.76 ± 12.65	60.59 ± 7.35 (*n* = 49)	3.63 ± 4.42 (*n* = 46)	29.23 ± 42.48 (*n* = 54)	/
*p*‐value		0.009		0.03*	0.04*	0.12	< 0.0001	/

	Muscle tissue homogenate analysis							
	*n*	Sex		Age (years)	Modified MRC sum score	Disease duration (years)	CK (μkat/L)	IBMFRS
		M	F					
IBM	17	10	7	67.18 ± 9.77	57.27 ± 8.83 (*n* = 15)	4.69 ± 5.70 (*n* = 16)	9.38 ± 4.08 (*n* = 16)	/
IIM	19	8	11	60.68 ± 10.58	60.06 ± 6.62 (*n* = 17)	3.61 ± 5.98 (*n* = 18)	70.71 ± 71.49 (*n* = 18)	/
IMNM	7	5	2	64.29 ± 8.90	54.86 ± 4.30	3.14 ± 7.45	99.32 ± 58.48	/
OLM	6	2	4	57.50 ± 15.81	62.60 ± 4.78 (*n* = 5)	2.83 ± 4.07	46.07 ± 58.30	/
PM‐M	4	0	4	62.75 ± 1.50	65.00 ± 7.39	6.50 ± 6.86	16.17 ± 14.35	/
DM	2	1	1	53.50 ± 3.54	64 (*n* = 1)	0 (*n* = 1)	236.4 (*n* = 1)	/
HER	5	1	4	43.60 ± 18.64	66.00 ± 5.66 (*n* = 4)	7.00 ± 8.89 (*n* = 3)	6.62 ± 3.76	/
DM2	2	0	2	50.00 ± 5.66	70 (*n* = 1)	2.00 ± 2.83	6.89 ± 5.21	/
GSD5	3	1	2	39.33 ± 24.70	64.67 ± 6.11	17 (*n* = 1)	6.44 ± 3.81	/
CTR	2	2	0	53.00 ± 15.56	66.50 ± 4.95	/	2.32 ± 1.73	/
Total	43	21	22	60.91 ± 13.33	59.92 ± 7.80 (*n* = 38)	4.35 ± 5.98 (*n* = 37)	35.62 ± 56.30 (*n* = 41)	/
*p*‐value		0.19		0.01	0.09	0.29	< 0.0001	/

	Serum analysis							
	*n*	Sex		Age (years)	Modified MRC sum score	Disease duration (years)	CK (μkat/L)	IBMFRS
		M	F					
IBM	11	9	2	64.64 ± 8.95	52.13 ± 11.99 (*n* = 8)	8.27 ± 5.39	7.18 ± 5.38 (*n* = 10)	24.00 ± 6.39 (*n* = 10)
IIM	25	9	16	61.96 ± 13.04	62.07 ± 6.73 (*n* = 15)	6.88 ± 6.82 (*n* = 24)	17.22 ± 35.58 (*n* = 24)	/
IMNM	11	7	4	65.00 ± 8.45	59.14 ± 7.93 (*n* = 7)	4.18 ± 5.93	17.21 ± 23.15 (*n* = 10)	/
OLM	6	1	5	61.83 ± 13.11	64.00 ± 6.33 (*n* = 4)	8.50 ± 2.59	4.71 ± 3.47	/
PM‐M	5	0	5	65.00 ± 14.97	65.67 ± 3.22 (*n* = 3)	13.20 ± 8.96	3.37 ± 1.89	/
DM	3	1	2	46.00 ± 18.73	64 (*n* = 1)	1.0 ± 0 (*n* = 2)	65.39 ± 87.63	/
HER	53	18	35	53.91 ± 13.31	60.28 ± 8.69 (*n* = 36)	21.44 ± 13.27 (*n* = 39)	6.24 ± 6.81 (*n* = 52)	/
DM1	6	5	1	41.33 ± 12.75	55.67 ± 16.80	8.0 ± 7.94 (*n* = 3)	3.95 ± 1.35 (*n* = 5)	/
DM2	18	3	15	60.50 ± 9.45	61.70 ± 5.33 (*n* = 10)	18.54 ± 8.76 (*n* = 13)	5.65 ± 4.31	/
FSHD	5	3	2	52.00 ± 8.00	60.0 ± 2.0 (*n* = 3)	21.0 ± 14.7 (*n* = 4)	6.70 ± 2.72	/
GSD2	18	7	11	51.33 ± 14.08	60.47 ± 7.17 (*n* = 15)	26.24 ± 15.71 (*n* = 17)	3.99 ± 2.62	/
GSD5	6	0	6	56.00 ± 16.53	66.0 ± 5.66 (*n* = 2)	20.50 ± 0.71 (*n* = 2)	16.23 ± 15.33	/
MIT	30	9	21	51.80 ± 18.73	67.67 ± 4.80 (*n* = 6)	25.20 ± 12.84 (*n* = 20)	4.05 ± 4.20 (*n* = 29)	/
sDel	19	5	14	57.47 ± 14.69	65.33 ± 6.43 (*n* = 3)	31.08 ± 10.44 (*n* = 13)	5.19 ± 4.95 (*n* = 18)	/
pMut	11	4	7	42.00 ± 21.50	70 ± 0 (*n* = 3)	14.29 ± 9.50 (*n* = 7)	2.17 ± 1.24	/
CTR	37	17	20	50.84 ± 18.11	70 ± 0 (*n* = 27)	/	2.25 ± 1.87	/
Total	156	62	94	54.82 ± 15.93	63.20 ± 8.82 (*n* = 92)	16.98 ± 13.36 (*n* = 94)	6.64 ± 15.43 (*n* = 152)	24.00 ± 6.39 (*n* = 10)
*p*‐value		0.03		0.01*	< 0.0001	< 0.0001	< 0.0001	/

* not significant in Dunn's post hoc test.

Abbreviations: CAM, chloroquine‐associated myopathy; CK, creatine kinase; CTR, control samples; DM, dermatomyositis; DM1, myotonic dystrophy type 1; DM2, myotonic dystrophy type 2; FLNC, filamin C; FSHD, facioscapulohumeral muscular dystrophy; GSD2, glycogen storage disease 2; GSD5, glycogen storage disease 5; HER, hereditary myopathies; IBM, inclusion body myositis; IIM, idiopathic inflammatory myopathies; IBMFRS, inclusion body myositis functional rating scale; IMNM, immune‐mediated necrotising myopathy; LDB3, LIM domain binding 3; MIT, mitochondrial myopathies; MFM, myofibrillar myopathy; modified MRC sum score, modified Medical Research Council sum score; MPD, distal myopathy; NEUR, neurogenic myopathy; non‐VAC, other myopathies without vacuoles; OLM, overlap myositis; OM, other myopathies; PM‐M, polymyositis with mitochondrial pathology; pMut, point mutations of mitochondrial DNA; sDel, singular deletions of mitochondrial DNA; SLONM, sporadic late‐onset nemaline myopathy; SQSTM1, sequestosome 1; TIA1, Tia1 cytotoxic granule–associated RNA binding protein; VAC (rim.), other myopathies with rimmed vacuoles; VAC (non‐rim.), other myopathies without rimmed vacuoles.

For immunohistochemical analysis, a total of 63 biopsy specimens were included. We compared samples from patients diagnosed with IBM (*N* = 19) and those from patients diagnosed with other neuromuscular diseases (NMDs). These diseases were categorised into two groups: idiopathic inflammatory myopathies other than IBM (IIM, *N* = 21, 9 immune‐mediated necrotising myopathy [IMNM], 6 overlap myositis [OLM], 4 polymyositis with mitochondrial pathology [PM‐Mito] and 2 dermatomyositis [DM]) and a cohort of other myopathies (OM), comprising both non‐vacuolated (non‐VAC, *N* = 3, all myotonic dystrophy Type 2) and vacuolated (VAC) myopathies. The latter group was further stratified into OM with rimmed vacuoles, some of which also exhibited mild inflammatory features (VAC [rim.], *N* = 9, 3 LDB3‐associated myopathy, 1 FLNC‐associated myopathy, 1 TIA1/SQSTM1‐associated myopathy, 1 SQSTM1‐multisystem proteinopathy, 1 myofibrillar myopathy, 1 distal myopathy and 1 paraspinal denervation) considered potential IBM mimics and OM with non–rimmed vacuoles (VAC [non‐rim.], *N* = 8, 3 Pompe's disease, 3 McArdle's disease, 1 chloroquine myopathy and 1 sporadic late‐onset nemaline myopathy). Healthy patients without any neurological or NMD served as control group (CTR, *N* = 3).

For homogenate analysis, IBM samples (*N* = 17) were compared to IIM (*N* = 19, 7 IMNM, 6 OLM, 4 PM‐Mito and 2 DM) and a cohort of hereditary myopathies (HER, *N* = 5, 2 myotonic dystrophy Type 2 and 3 McArdle's disease) and CTR (*N* = 2).

Furthermore, a total of 156 serum samples were investigated, including 11 IBM, 25 IIM (11 IMNM, 6 OLM, 5 PM‐Mito and 3 DM), 53 HER (18 MATR3‐associated myopathy, 6 myotonic dystrophy Type 1, 18 myotonic dystrophy Type 2, 5 facioscapulohumeral muscular dystrophy and 6 McArdle's disease) and 30 mitochondriopathy (MIT) patients, as well as 37 sera of healthy volunteers (CTR).

Correct classification was confirmed by a neurologist with extensive experience in the diagnosis and treatment of neuromuscular disorders (AM), who critically appraised the classification against the respective current diagnostic criteria. During the conception and conduction of the current study, the ENMC 2011 diagnostic criteria for IBM were valid and were used for patient identification and categorisation [46]. Recently, the ENMC working group published updated diagnostic criteria [47]. While all included patients also meet the updated diagnostic criteria, some of the subcategories used in the present study (i.e., clinicopathologically defined IBM, clinically defined IBM and probable IBM) are no longer included in the current diagnostic criteria.

In addition to biological samples, demographic, clinical and paraclinical data such as age, sex, disease duration, modified Medical Research Council sum score (MRC‐SS), IBM functional rating scale (IBMFRS), anti–cN1A antibody status and creatine kinase (CK) activity were collected from patients' records. The MRC‐SS was modified by including finger flexion as a typical symptom of IBM to account for clinical severity, resulting in a maximum MRC‐SS of 70 [48].

This study was approved by the ethical committee of Martin Luther University of Halle‐Wittenberg (vote no. 2021‐101). All subjects included in this study gave their informed consent.

### 2.2 (Immuno‐)histochemical Staining

Staining with haematoxylin eosin (HE) and, in selected samples, for Gömöri trichrome (TR), nicotinamide adenine dinucleotide (NADH), periodic acid–Schiff (PAS), succinic dehydrogenase (SDH) and acid phosphatase (AP) were performed on 5‐μm sections according to the standard procedures of the facility. The staining protocols are available upon reasonable request by contacting the authors. For the immunohistochemical staining, sections were fixed in formaldehyde solution (4%), rinsed in tris‐buffered saline (TBS), incubated in hydrogen peroxide solution (3%), rinsed again and treated with blocking solution (ZytoChem‐Plus HRP polymer kit, Zytomed Systems, Germany). Mouse monoclonal anti–αSN primary antibody LB509 (Santa Cruz Biotechnology, United States) was added, followed by rinsing and incubation with a post–blocking reagent and HRP‐conjugated secondary antibodies. Finally, an AEC substrate kit (Zytomed Systems, Germany) was used for visualisation, followed by counterstaining with haemalum solution. Samples without primary antibodies (replaced with TBS) served as negative controls. Detailed information on the chemicals and antibodies used can be found in Table S1.

### 2.3 Histopathological Evaluation and Quantitative Assessment of αSN Immunoreactivity

Histopathologic evaluation of stained specimens was performed by an experienced neuropathologist (GS‐D) together with a doctoral researcher trained in muscle pathology (TM), both blinded to clinical data. Samples were digitised and then analysed using ImageJ software and the bio‐formats plugin [49, 50]. To ensure that the data were representative, approximately 350 muscle fibres were examined in each biopsy by analysing all fibres within the range of a digital high‐power field (approximately 350 μm in width) along the horizontal and vertical axes (representative analyses in Figure S1). Each fibre was assigned to one or more of the following categories, if applicable: vacuole‐containing, atrophic, regenerating and necrotic fibres with sarcoplasmic reactivity to αSN or fibres with sarcolemmal reactivity to αSN. Vacuoles included both RVs and non‐RVs as determined on HE staining. A fibre was defined as atrophic if the width perpendicular to its maximum diameter was less than 20 μm. Regenerating fibres were identified based on typical morphology (basophilic cytoplasm and enlarged vesicular internal nuclei with prominent nucleoli).

### 2.4 Preparation of Muscle Tissue Homogenates

Appropriate muscle biopsy samples were thoroughly rinsed in phosphate‐buffered saline, minced and homogenised on ice using Dounce homogenisers in a modified extraction buffer (MEB) comprising the extraction buffer from the human αSN ELISA kit (Abcam, United Kingdom), Halt protease inhibitor cocktail and 0.5‐M EDTA solution (both diluted 1:100; Thermo Fisher Scientific, United States). Lysates were incubated on ice for 20 min followed by centrifugation at 16,100 *g* and 5°C for 15 min. Supernatants were transferred to fresh tubes and immediately frozen at −80°C until use.

### 2.5 Protein Determination

Protein concentration in muscle tissue homogenates was determined using the Pierce BCA protein assay kit (Thermo Fisher Scientific, United States) according to the internal laboratory protocol. Briefly, homogenates were incubated with 50‐mM NaOH solution at room temperature for 1 day and transferred to a 96‐well plate the next day together with bovine serum albumin solutions as standards and NaOH solution as blank. After adding 200 μL of BCA working reagent to each well, the plate was incubated at 350 rpm and 60°C for 45 min, and reactivity was measured photometrically at 560 nm.

### 2.6 Determination of αSN Concentration in Muscle and Serum Samples

αSN concentration was determined using the human αSN ELISA kit (Abcam, United Kingdom) for tissue samples and the αSN ELISA kit (EUROIMMUN, Germany) for serum samples according to the manufacturer's instructions. Muscle tissue homogenates were appropriately diluted to the same total protein concentration beforehand.

### 2.7 Determination of Haemoglobin Concentration in Muscle and Serum Samples

Given the expression of αSN in red blood cells as a peripheral source of αSN, haemoglobin (Hb) concentrations in muscle and serum samples were quantified to determine the influence of haemolysis [51]. In muscle tissue homogenates, Hb concentration was determined photometrically using an Hb stock solution as standard and MEB as blank [52]. Extinction was measured at 414 nm in the Nanodrop OneC system (Thermo Fisher Scientific, United States). In serum samples (*n* = 124), the concentration was quantified using the human Hb ELISA kit (Abcam, United Kingdom) according to the manufacturer's protocol.

### 2.8 Statistical Analysis

Statistical analysis was performed using GraphPad Prism 8.3.0 (GraphPad Software, United States). The data were tested for normal distribution using the Kolmogorov–Smirnov test and graphically using Q–Q plots. As most of the data were not normally distributed, the non–parametric Kruskal–Wallis test with Dunn's post hoc test and Wilcoxon–Mann–Whitney test were used to compare continuous variables, as appropriate. Correlations were analysed using Spearman's rank coefficient. Uni‐ and multivariable linear and logistic regression models were built to investigate the associations of variables. Receiver operating characteristic (ROC) analysis was used to determine the diagnostic performance. Best cutoff values were calculated by maximising the Youden index [53]. Results were considered significant when the *p*‐value was < 0.05. Unless otherwise stated, all values are presented as mean ± one standard deviation (SD).

## 3 Results

### 3.1 Characteristics of the Study Cohort

Demographic and clinical characteristics of the study population are summarised in Table 1. A total of 21 patients with IBM were included (16 clinicopathologically defined, 3 clinically defined and 2 probable). The cohort consisted of 14 males (67%) and seven females (33%), resulting in a male‐to‐female ratio of 2:1. For 11 patients, serum samples and further clinical data were available. Among these, the median ages at symptom onset and diagnosis were 55 (45–73) and 58 (46–76) years, respectively, resulting in a calculated median diagnostic delay of 2 (0–7) years. The mean IBMFRS was 24 (± 6.39), with missing information for one patient. Five patients tested positive for anti–cN1A autoantibodies, while four were classified as seronegative (two patients with missing antibody status).

While Kruskal–Wallis analysis suggested significant between‐group differences with regard to age, Dunn's post hoc test found no difference in the cohorts used for histological and serum analyses. Only in the cohort used for tissue homogenates were IBM patients significantly older than HER patients (*p* = 0.01). There were significant differences in gender distribution in the serum cohort (*p* = 0.03). Modified MRC‐SS and disease duration did not differ significantly between the groups included in the muscle biopsy analyses when analysed with Dunn's post hoc test. In the serum cohort, modified MRC‐SS was significantly lower in IBM (52.13 ± 11.99), IIM (62.07 ± 6.73) and HER (60.28 ± 8.69) compared to healthy controls (70 ± 0, *p* < 0.001). In addition, disease duration was significantly longer in MIT (25.20 ± 12.84 years) and HER (21.44 ± 13.27 years) patients compared to IBM (8.27 ± 5.39 years, *p* < 0.02) and IIM (6.88 ± 6.82 years, *p* < 0.0001) patients. IIM patients had higher CK activity than all other groups. Because of the retrospective study design, there was a lack of clinical and paraclinical data for some patients.

### 3.2 Allocation of αSN Immunoreactivity

**FIGURE 1:**
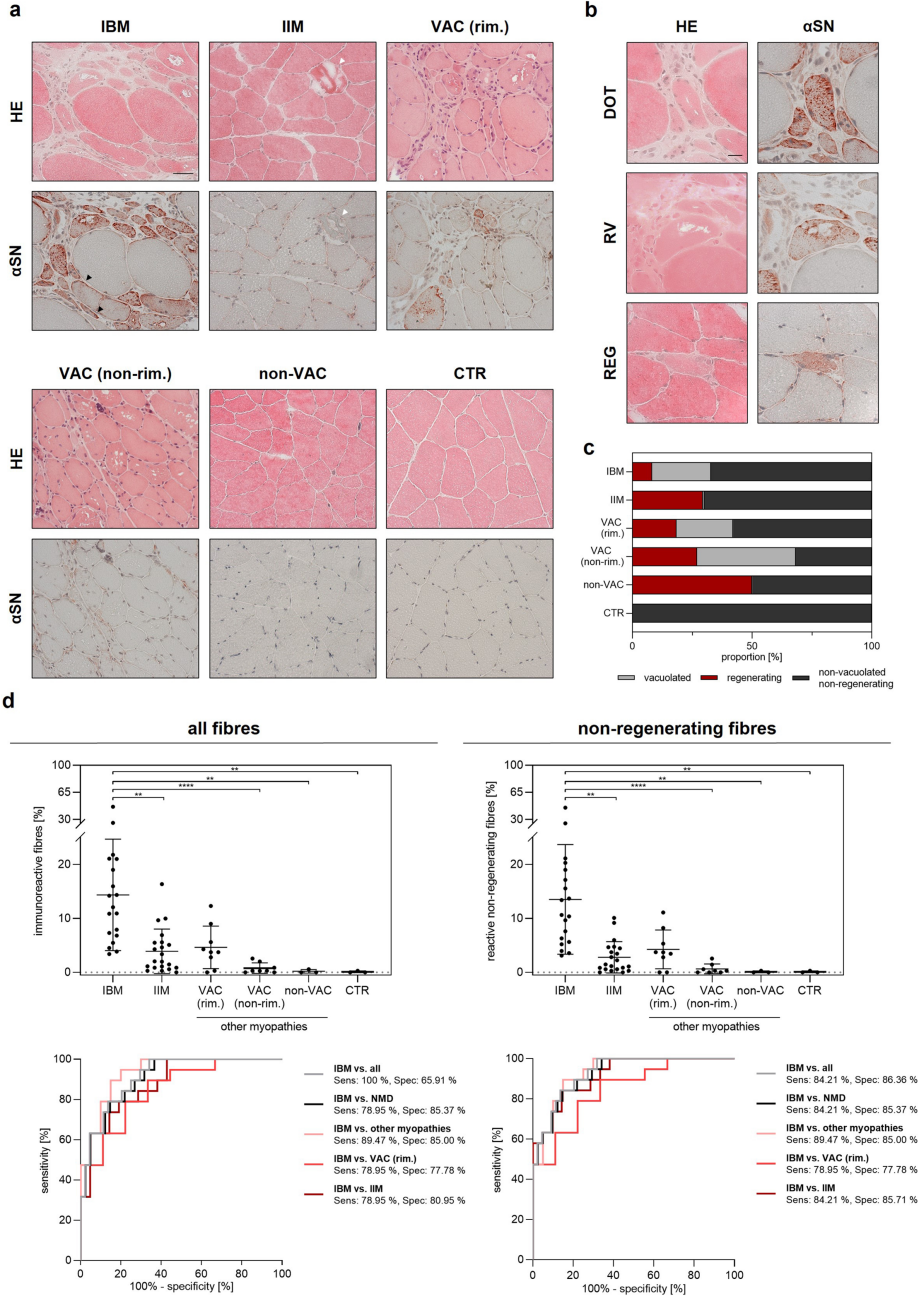
Distribution and quantification of αSN immunoreactivity. (a) HE and αSN staining. IBM samples exhibited strong immunoreactivity for αSN, while only single fibres were positive in other forms of IIM and myopathies with rimmed vacuoles. Note the subsarcolemmal enhancement of some fibres in IBM (black arrowheads). Necrotic fibres did not show reactivity (white arrowhead). (b) Allocation of αSN reactivity in different fibre types. Most fibres in IBM showed small dotty inclusions (DOT), while less pronounced, homogeneous reactivity (REG) was found in most regenerating fibres. No reactivity was observed within rimmed vacuoles (RV). (c) Nature of immunoreactive fibres in different groups. In samples other than IBM, regenerating fibres accounted for a larger proportion of immunoreactive fibres. (d) Quantification of αSN immunoreactivity and ROC analysis to differentiate IBM from the other subgroups studied. All fibres: Quantification revealed a significantly higher proportion of reactive fibres (%) in IBM samples, while ROC analysis shows a good differentiation of IBM from other IIMs and other myopathies. Non–regenerating fibres: Quantification of αSN immunoreactivity only in non–regenerating fibres improved discrimination of IBM from other IIMs. The discrimination of IBM from other myopathies in general and myopathies with rimmed vacuoles did not differ after excluding regenerating fibres. The ROC analyses of IBM versus VAC (non‐rim.), non‐VAC and CTR yielded a sensitivity and specificity of 100%, respectively. These results are not displayed here to enhance clarity. *p* < 0.05*, *p* < 0.01**, *p* < 0.001*** and *p* < 0.0001****. Abbreviations: all, all specimens other than IBM; CTR, control specimens; DOT, dotty αSN immunoreactivity; HE, haematoxylin eosin staining; IBM, inclusion body myositis; IIM, idiopathic inflammatory myopathies; NMD, neuromuscular diseases (including IIM and other myopathies); REG, regenerating muscle fibre; RV, rimmed vacuole; VAC (rim.), vacuolar myopathies with rimmed vacuoles; VAC (non‐rim.), vacuolar myopathies with non–rimmed vacuoles; non‐VAC, other myopathies without vacuoles; αSN, α‐synuclein staining; scale bars: (a) 50 μm and (b) 20 μm.

Representative examples of αSN staining in patients with NMDs and CTR are illustrated in Figure 1a. All biopsy samples from IBM patients showed immunoreactivity for αSN, mostly seen as multiple small punctate foci throughout the whole fibre (Figure 1b, DOT). In addition, some fibres showed reactivity as a slight subsarcolemmal enhancement of staining (Figure 1a, black arrowhead), while serial sections (Figure S2) did not show a clear association with lipofuscin (AP staining), glycogen (PAS staining) or mitochondria (Gömöri trichrome and SDH stains). Although reactivity was occasionally observed to be stronger adjacent to RVs, it was never found to be localised within the vacuoles (Figure 1b, RV). In regenerating fibres, a moderate and mostly homogeneous reactivity was observed (Figure 1b, REG). No αSN reactivity was found in necrotic fibres (Figure 1a, white arrowhead). CTR samples did not contain any vacuolar, necrotic or regenerating fibres. Two out of the three samples did not show any sarcoplasmic reactivity. However, in one sample, two atrophic fibres were identified, one of which showed slight reactivity.

### 3.3 Quantification of the Histological Findings and αSN Immunoreactivity

Detailed results of the quantitative analysis of αSN immunoreactivity are shown in Table 2. The proportion of fibres immunoreactive for αSN (Figure 1d, all fibres) was significantly higher in IBM (14.35% ± 10.32%) compared to CTR (0.09% [± 0.16%], *p* = 0.0020), IIM (3.90% [± 4.13%], *p* = 0.0053), VAC (non‐rim.) (0.78% [± 0.97%], *p* < 0.0001) and non‐VAC (0.19% [± 0.33%], *p* = 0.0035). While the proportion of immunoreactive fibres also tended to be lower in VAC (rim.), this did not reach significance (4.63% [± 3.94%], *p* = 0.197). Among IIM samples, polymyositis with mitochondrial pathology (PM‐Mito, 6.25% ± 4.31%) showed the highest amount of immunoreactive fibres. However, there was no significant difference compared to other groups (IMNM: 4.53% [± 4.85%]; OLM: 2.44% [± 2.76%]; and DM: 0.68% [± 0.57%]; *p* > 0.05).

**TABLE 2:** Detailed results of quantification analysis.

Category	IBM	IIM	VAC (rim.)	VAC (non‐rim.)	Non‐VAC	CTR	*p*
*n*	19	21	9	8	3	3	
FIB tot. (*n*)	352.2 ± 13.72	351.2 ± 7.92	346.9 ± 11.02	357.5 ± 12.56	354.0 ± 9.85	359.7 ± 11.06	0.4867
ATR tot./FIB tot. (%)	15.05 ± 10.31	5.44 ± 7.52	18.23 ± 24.28	7.89 ± 15.22	0.47 ± 0.33	0.18 ± 0.32	0.0003
VAC tot./FIB tot. (%)	4.42 ± 4.21*	0.20 ± 0.35	3.47 ± 3.39	4.96 ± 4.96	0.09 ± 0.16	0	< 0.0001
REG tot./FIB tot. (%)	1.09 ± 0.80	1.32 ± 1.91	0.51 ± 0.63	0.14 ± 0.21	0.09 ± 0.16	0	0.0005
NEC tot./FIB tot. (%)	0.02 ± 0.07	0.36 ± 0.70	0.57 ± 0.82	0.11 ± 0.15	0	0	0.0081
FIB reac./FIB tot. (%)	14.35 ± 10.32	3.89 ± 4.13	4.63 ± 3.94	0.78 ± 0.97	0.19 ± 0.33	0.09 ± 0.16	< 0.0001
VAC reac./VAC tot. (%)	71.46 ± 20.32*	19.45 ± 30.58	39.96 ± 34.00	17.52 ± 34.19	0	/	0.0012
ATR reac./ATR tot. (%)	40.08 ± 17.10	29.05 ± 23.98	24.73 ± 26.07	17.51 ± 22.75	11.11 ± 19.24	50	0.0849
REG reac./REG tot. (%)	93.50 ± 13.77	94.37 ± 8.26	86.90 ± 21.39	75.00 ± 35.36	100	/	0.8001
FSLR/FIB tot. (%)	17.07 ± 12.45	8.67 ± 6.37	7.13 ± 6.56	4.64 ± 3.62	0.18 ± 0.32	2.59 ± 3.04	0.0002
VAC reac./FIB reac. (%)	24.55 ± 16.63*	0.61 ± 2.37	23.64 ± 18.63	41.27 ± 47.11	0	0	< 0.0001
ATR reac./FIB reac. (%)	40.68 ± 19.24	33.57 ± 35.05	48.53 ± 32.83	63.76 ± 37.77	50	100	0.1677
REG reac./FIB reac. (%)	8.25 ± 5.52	29.47 ± 26.37	18.45 ± 33.31	26.98 ± 38.88	50	0	0.0483

* *n* = 18.

Abbreviations: ATR, atrophic fibres; CTR, control specimens; FIB, fibres; FSLR, fibres with sarcolemmal enhancement of immunoreactivity; IBM, inclusion body myositis; IIM, idiopathic inflammatory myopathies; NEC, necrotic fibres; non‐VAC, other myopathies without vacuoles; reac., immunoreactive fibres; REG, regenerating fibres; tot., total; VAC, vacuolated fibres; VAC (non‐rim.), other myopathies without rimmed vacuoles; VAC (rim.), other myopathies with rimmed vacuoles.

In general, there was no obvious difference in the staining pattern in subgroups with relevant αSN reactivity (IBM, IIM and VAC [rim.]). Most fibres showed punctate distribution throughout the cytoplasm, as exemplified in Figure S3 (IBM vs. IMNM). The highest amount of subsarcolemmal reactivity was observed in IBM samples (17.07% ± 12.45%) as compared to the other groups (IIM: 8.67% [± 6.37%], *p* = 0.23; VAC [rim.]: 7.13% [± 6.56%], *p* = 0.19; VAC [non‐rim.]: 4.62% [± 3.62%], *p* = 0.02; non‐VAC: 0.18% [± 0.32%], *p* = 0.002; and CTR: 2.59% [± 3.04%], *p* = 0.07). The highest number of atrophic fibres was found in IBM (15.05% ± 10.31%) and in VAC (rim.) (18.23% ± 24.28%), while there was no significant difference in reactivity between the groups. Necrotic fibres were most prevalent in IIM (0.30% ± 0.65%) and VAC (rim.) (0.57% ± 0.82%).

Irrespective of the group analysed, αSN immunoreactivity occurred in the vast majority of regenerating muscle fibres (92.22% ± 14.39%), with regenerating fibres accounting for an important proportion of total αSN‐immunoreactive fibres in IIM (29% [± 26%]), VAC (rim.) (18% [± 33%]), VAC (non‐rim.) (27% [± 39%]) and non‐VAC (50% [± 0%]; Figure 1c). Exclusion of all regenerating fibres (Figure 1d, non–regenerating fibres) resulted in an increased difference in reactivity between IBM (13.51% ± 10.14%) and other groups (IIM: 2.78% [± 2.90%], *p* = 0.0011; VAC [rim.]: 4.26% [± 3.59%], *p* = 0.17; VAC [non‐rim.]: 0.64% [± 0.91%], *p* < 0.0001; and non‐VAC: 0.10% [± 0.17%], *p* = 0.004).

There was a significant correlation between αSN‐immunoreactive fibres and other quantitative histological parameters such as total atrophic fibres [%] (*r* = 0.68, *p* < 0.001), total regenerating fibres [%] (*r* = 0.72, *p* < 0.001) and reactive vacuolated fibres [%] (*r* = 0.56, *p* = 0.016) (Figure S4). However, there was no significant correlation of αSN‐immunoreactive fibres (total or non‐regenerating) with clinical and paraclinical parameters including age, disease duration, modified MRC‐SS and CK.

### 3.4 Differentiation of IBM From Other Subgroups of Neuromuscular Diseases by αSN Reactivity

To assess the diagnostic value of αSN immunostaining in distinguishing IBM from other conditions, ROC analyses were conducted, with detailed results outlined in Table 3. When all fibres were considered, αSN immunostaining differentiated IBM from all NMDs studied with a sensitivity of 78.95% and a specificity of 85.37% (AUC = 0.91; cutoff > 6.215%). This accuracy improved when only non–regenerating fibres were analysed, yielding a sensitivity of 84.21% with the same specificity (AUC = 0.93; cutoff > 4.96%).

**TABLE 3:** Detailed results of ROC analyses with different quantified histological parameters.

Parameter	Analysis	AUC	95% CI	*p*	Cutof*f*	Sensitivity (%)	95% CI	Specificity (%)	95% CI
All fibres (%)	IBM vs. all	0.91	0.84–0.98	< 0.0001	> 3.365	100	83.18–100	65.91	51.14–78.12
	IBM vs. NMD	0.91	0.83–0.98	< 0.0001	> 6.215	78.95	56.67–91.49	85.37	71.56–93.12
	IBM vs. other myopathies	0.93	0.86–1.00	< 0.0001	> 4.39	89.47	68.61–98.13	85	63.96–94.76
	IBM vs. VAC (rim.)	0.85	0.71–1.00	0.0029	> 6.215	78.95	56.67–91.49	77.78	45.26–96.05
	IBM vs. VAC (non‐rim.)	1.00	1.00–1.00	<0.0001	> 2.97	100	83.18–100	100	67.56–100
	IBM vs. non‐VAC	1.00	1.00–1.00	0.0064	> 1.975	100	83.18–100	100	43.85–100
	IBM vs. IIM	0.88	0.78–0.98	< 0.0001	> 6.19	78.95	56.67–91.49	80.95	60.00–92.33
	IBM vs. CTR	1.00	1.00–1.00	0.0064	> 1.83	100	83.18–100	100	43.85–100
Non–regenerating fibres (%)	IBM vs. all	0.93	0.87–0.99	< 0.0001	> 4.96	84.21	62.43–94.48	86.36	73.29–93.60
	IBM vs. NMD	0.93	0.86–0.99	< 0.0001	> 4.96	84.21	62.43–94.48	85.37	71.56–93.12
	IBM vs. other myopathies	0.93	0.86–1.00	< 0.0001	> 3.855	89.47	68.61–98.13	85	63.96–94.76
	IBM vs. VAC (rim.)	0.85	0.70–1.00	0.0034	> 5.475	78.95	56.67–91.49	77.78	45.26–96.05
	IBM vs. VAC (non‐rim.)	1.00	1.00–1.00	<0.0001	> 2.835	100	83.18–100	100	67.56–100
	IBM vs. non‐VAC	1.00	1.00–1.00	0.0064	> 1.7	100	83.18–100	100	43.85–100
	IBM vs. IIM	0.92	0.84–1.00	<0.0001	> 4.96	84.21	62.43–94.48	85.71	65.36–95.02
	IBM vs. CTR	1.00	1.00–1.00	0.0064	> 1.70	100	83.18–100	100	43.85–100

Abbreviations: CTR, control specimens; IBM, inclusion body myositis; IIM, idiopathic inflammatory myopathies; NMD, neuromuscular diseases beyond IBM; VAC (rim.), other myopathies with rimmed vacuoles; VAC (non‐rim.), other myopathies without rimmed vacuoles; non‐VAC, other myopathies without vacuoles.

In distinguishing IBM from different NMD subgroups, CTR, VAC (non‐rim) and non‐VAC were delineated with virtually perfect accuracy (100% sensitivity and specificity, AUC = 1.00). The separation of IBM from IIM (Figure 1d, all fibres) showed a sensitivity of 78.95% and a specificity of 80.95% (AUC = 0.88; cutoff > 6.19%). Excluding regenerating fibres (Figure 1d, non–regenerating fibres) further improved diagnostic performance, with sensitivity rising to 84.21%, specificity to 85.71% and AUC to 0.92 (cutoff > 4.96%). Myopathies with rimmed vacuoles (VAC [rim.]) could be differentiated from IBM with a sensitivity of 78.95% and a specificity of 77.78% (AUC = 0.85; cutoff > 6.215%) when analysing all fibres, with no improvement seen after excluding regenerating fibres.

### 3.5 αSN Serum Concentrations

Serum Hb concentrations were used as a surrogate for haemolysis. While no clear correlation between Hb and αSN was seen in the graphical analysis (Figure S5a), a correlation was suggested by statistical analysis in the diseased cohort (*r* = 0.525). However, Hb concentration was generally negligible in most serum samples, with no significant differences between the groups (*p* = 0.63). Furthermore, no correlation was observed in CTR. Therefore, no adjustment for haemolysis was applied. In healthy individuals, αSN concentration correlated significantly with age (*r* = 0.41, *p* = 0.01) and CK (*r* = 0.39, *p* = 0.02). While simple linear regression analyses suggested a relevant effect, multiple linear regression revealed no significant influence on αSN concentration for either parameter (Table S2).

**FIGURE 2:**
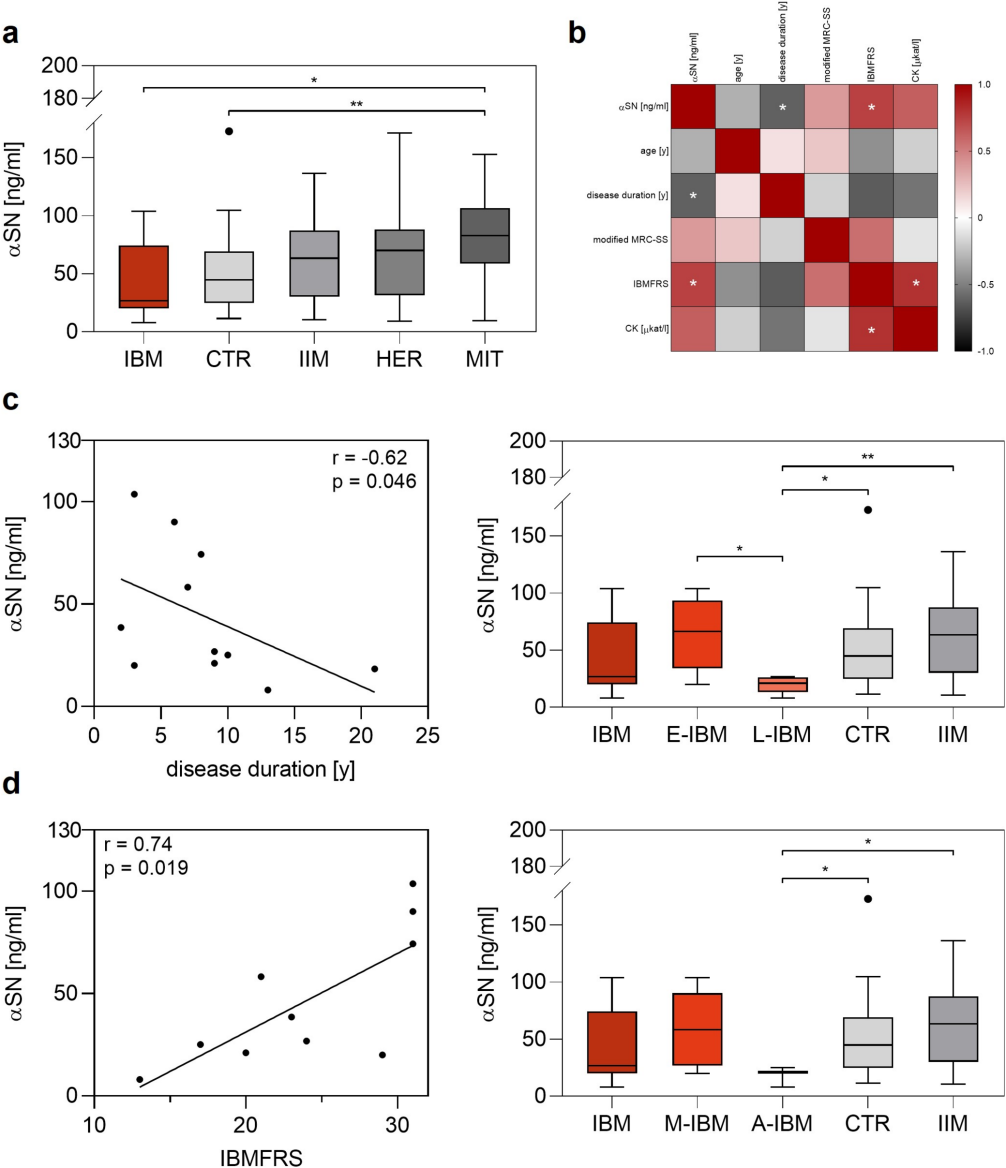
Analysis of αSN concentration in serum samples. (a) IBM samples tended to have lower serum αSN concentration than other groups (left side, notice median), although not significant. (b) Correlation of serum αSN concentration with different epidemiological and clinical parameters. Serum αSN concentration correlated significantly with patients' disease duration and IBMFRS. IBMFRS correlated significantly with CK activity. (c) Correlation analysis of αSN with patients' disease duration (left) and stratification of IBM patients in an early (≤ 8 years disease duration) and a late‐stage (> 8 years disease duration) IBM (right). (d) Correlation analysis of αSN with patients' IBMFRS (left) and stratification in mild (IBMFRS > 20) and advanced (IBMFRS ≤ 20) IBM (right side). Abbreviations: A‐IBM, advanced IBM; CK, creatine kinase; CTR, control samples; E‐IBM, early‐stage IBM; HER, hereditary myopathies; IBMFRS, inclusion body myositis functional rating scale; IBM, inclusion body myositis; IIM, idiopathic inflammatory myopathies; L‐IBM, late‐stage IBM; M‐IBM, mild IBM; MIT, mitochondrial myopathies; modified MRC sum score, modified Medical Research Council sum score; αSN, α‐synuclein.

Although there was a trend towards lower αSN concentrations in IBM (44.03 ± 32.47 ng/mL), the difference was not significant compared to CTR (50.47 ± 34.12 ng/mL, *p* = 0.53; Figure 2a). However, correlation analyses in IBM (Figure 2b) revealed a significant correlation of αSN concentrations with patients' disease duration (*r* = −0.62, *p* = 0.046, Figure 2b) and IBMFRS (*r* = 0.74, *p* = 0.019, Figure 2c). Stratification for an early (E‐IBM, ≤ 8 years disease duration, *n* = 6; αSN = 64.16 ± 31.52 ng/mL) and a late stage (L‐IBM, > 8 years disease duration, *n* = 5; αSN = 19.86 ± 7.45 ng/mL) showed a significantly lower αSN concentration in L‐IBM compared to CTR (*p* = 0.018), IIM (*p* = 0.0045) and E‐IBM (*p* = 0.03, Mann–Whitney test used for all, L‐IBM versus IIM also significant in Kruskal–Wallis test, Figure 2c). Furthermore, after categorisation into mild (M‐IBM, IBMFRS > 20, *n* = 7, αSN = 58.83 ± 32.04 ng/mL) and advanced (A‐IBM, IBMFRS ≤ 20, *n* = 3, αSN = 18.05 ± 8.97 ng/mL) IBM stages, there was a significantly lower αSN concentration in A‐IBM compared to CTR (*p* = 0.041) and IIM (*p* = 0.019, Mann–Whitney test used for both, not significant in Kruskal–Wallis test, Figure 2d).

Additionally, CK activity correlated significantly with IBMFRS (*r* = 0.803, *p* = 0.012) and showed a trend towards significant correlation with αSN concentrations (*r* = 0.62, *p* = 0.06, Figure 2b). No correlation of αSN concentrations with age or modified MRC‐SS was observed. There were no significant differences in αSN concentration with respect to ENMC 2011 categories, antibody status or gender.

### 3.6 αSN Concentrations in Muscle Tissue Homogenates

In tissue homogenates, Hb concentrations as a measure of haemolysis were significantly higher compared to serum samples (Figure S5b), with a significant correlation of αSN and Hb concentrations (*r* = 0.87, *p* < 0.0001, Figure S5c left). Visually, there was an exponential relationship between αSN and Hb, which was corrected by semi‐log transformation (Figure S5c right). However, no significant difference in the calculated log‐αSN/Hb ratio was observed between IBM (1.33 ± 0.54) and CTR (1.21 ± 0.22) nor between the other groups (IIM: 1.36 [± 0.31]; HER: 1.41 [± 0.45]) (*p* = 0.79, Kruskal–Wallis test, Figure S5d). Furthermore, multivariate logistic regression analysis revealed no significant associations between αSN and diagnosis (IBM vs. CTR) after accounting for Hb (AUC: 0.74 [95% CI: 0.34–1.00], *p* = 0.29). Correlation analysis did not show any significant association between log‐αSN/Hb ratios and (para‐)clinical parameters as well as quantified histological parameters in the whole cohort or IBM samples (data not shown).

## 4 Discussion

There is a need for valid biomarkers to support the diagnosis of IBM, especially in the early stages of the disease. In this respect, our study is the first to investigate the diagnostic ability of αSN to differentiate IBM from other neuromuscular disorders, particularly other forms of IIM and (hereditary) myopathies that show inflammation and rimmed vacuolar pathology and thus may mimic IBM. A multimodal assessment including histopathology, serum samples and tissue homogenates was used and correlated with clinical and paraclinical parameters of disease progression.

In the first approach, αSN immunostaining showed immunoreactivity for αSN in all IBM muscle samples, predominantly as multiple small punctate foci. This distribution is generally consistent with observations from two studies using the same antibody, whereas different or no reactivity has been reported for antibodies using other epitopes [7, 32, 44, 45]. This may suggest that the αSN aggregates observed in IBM may partly resemble those in synucleinopathies, given the similarities in the detected epitopes [54, 55]. In line with the findings of Askanas et al., αSN reactivity was also observed in regenerating muscle fibres, which significantly accounted for the total reactivity in IIM other than IBM [44]. The observed αSN reactivity in regenerating fibres may most likely be interpreted as a non–specific phenomenon in an immature fibre expressing a variety of proteins that are not found in the mature cell. Furthermore, subsarcolemmal αSN reactivity was found, which has previously been suggested to correspond to αSN at neuromuscular junctions [44]. However, the observed abundance of this phenomenon with a significantly higher occurrence in IBM questions this assumption. As the serial section histology used in this study did not show a clear association with lipofuscin, glycogen or accumulating mitochondria, the nature of this phenomenon remains elusive at this point. In contrast to previous reports, cytoplasmic reactivity was also observed in IIM samples other than IBM [44, 45].

Despite the striking difference in αSN reactivity between different variants of IIM and other myopathies, no previous study has focused on its diagnostic utility. Using a standardised immunoquantification method, a significant difference was found for IBM compared to IIM, non–rimmed vacuolar myopathies, non–vacuolar myopathies and CTR. Given the high reactivity for αSN in regenerating fibres, this difference was even more pronounced after their exclusion. While there was an apparent difference between IBM and myopathies with rimmed vacuoles (average αSN‐positive fibres 14.35% vs. 4.63%), significance was not reached, probably because of the small sample size. Consequently, ROC analysis on the ability to differentiate IBM from other neuromuscular disorders showed satisfactory sensitivity (78.95%) and specificity (85.37%). Again, sensitivity increased further (84.21%) when only non–regenerating fibres were assessed. Analyses of the various subgroups yielded analogous results, even in the group of myopathy with rimmed vacuoles as relevant IBM mimic (sensitivity 78.95% and specificity 77.78%). Several studies have reported highly variable sensitivity (12.5%–91%) and specificity (18%–100%) for the established immunopathological markers of protein accumulation p62 and TDP‐43 [32, 34, 36]. Therefore, the diagnostic accuracy of αSN may be at least similar. However, structured comparative analyses combining αSN, p62 and TDP‐43 are required to address this issue further.

The reason for αSN accumulation in IBM remains uncertain. Several studies have identified a variety of myopathic alterations (including dystrophic changes, inflammation, mitochondrial pathology, myofibrillar disorganisation and necrosis) in paraspinal muscle biopsies of patients with camptocormia due to Parkinson's disease, a prototype of a primary synucleinopathy [56, 57, 58, 59, 60]. However, to the best of our knowledge, αSN expression has not been studied in these biopsy specimens. Furthermore, only one study has described a rimmed vacuolar pathology in a subset of patients with Parkinson's disease [58]. Given the apparent occurrence of αSN reactivity in various myopathies with degenerative features, the observed αSN pathology may not point to a primary synucleinopathy but rather indicate a general sign of (primary or secondary) disturbance of proteostasis. This assumption may also apply to the currently used histological markers TDP‐43 and p62.

In Parkinson's disease and dementia with Lewy bodies, phosphorylation of αSN at serine residue 129 (pS129) has been identified as a characteristic feature of seeding‐competent αSN aggregates and is therefore considered a reliable biomarker for the diagnosis of PD and DLB. Whether S129 phosphorylation is a prerequisite for the formation of αSN aggregates remains controversial [61]. To the best of our knowledge, the phospho‐species of αSN have not been investigated in IBM. However, future studies of pS129–αSN in IBM may provide further insight into the relevance of αSN in the pathophysiology of the disease.

Notably, the highest αSN reactivity in IIMs other than IBM was found in patients diagnosed with PM‐Mito, although the difference compared to other IIMs was not statistically significant. PM‐Mito has widely been recognised as a precursor of IBM, but there remains an ongoing debate [62, 63, 64, 65]. Notably, previous studies have provided evidence that the p62 and TDP‐43 pathology observed in IBM is not present in PM‐Mito [34, 66]. Thus, it can be speculated that αSN reactivity may be of additional value in these early stages of IBM, which may not be adequately captured by the other established histological markers. However, comparative studies, including αSN, TDP‐43 and p62, together with clinical follow‐up data identifying patients who develop IBM during the course of the disease, are essential to provide substantial evidence for this hypothesis.

Although extensive quantification of reactive muscle fibres may not be practical in clinical routine, the increasing use of semi‐automated morphometric analysis may allow an approach as used in this study. Thus, the obtained results support a possible benefit of αSN immunostaining in the diagnostic routine of IBM. There was a significant correlation of αSN with other immunohistological signs of muscle degeneration (atrophic, vacuolated and regenerating muscle fibres) but not with demographic and clinical or paraclinical parameters of disease severity (e.g., disease duration, IBMFRS, MRC‐SS and CK). Thus, there is no evidence that histological analysis of αSN immunostaining may serve as a biomarker for IBM progression.

Given the apparent histological alteration of αSN, additional serum analyses were performed. To our knowledge, this is the first study to investigate serum αSN concentration in patients with IBM and a large cohort of NMDs. Serum αSN concentration tended to be lower in IBM compared to CTR, although no significance was reached. Therefore, serum αSN may not be suitable as a diagnostic biomarker for IBM. However, significant correlations were observed between αSN concentration and clinical markers of disease severity as measured by IBMFRS and disease duration. Consequently, stratification of IBM patients according to IBMFRS (mildly and severely affected) and disease duration (early and late stages) resulted in significantly lowered αSN concentrations in late stage as well as severely affected patients, respectively. In analogy to PD, where αSN was reported to correlate with the severity of motor symptoms and cognitive decline, αSN serum concentrations may serve as a monitoring biomarker for disease progression [67, 68]. This is particularly important as a blood‐based biomarker has not yet been established. However, to ensure the relevance of αSN dynamics at the individual patient level and thus evaluate the applicability of serum αSN as a monitoring biomarker, longitudinal analyses comparing clinical data and serum αSN concentration at different time points are mandatory.

The mechanism leading to the depletion of αSN in the peripheral blood of IBM patients can only be speculated. According to large tissue‐based proteome databases, αSN is not necessarily expressed in muscle tissue under physiological conditions, although RNA is detectable at low levels [69]. One study proposed that both monomeric and fibrillar αSN can be internalised into cells in vitro and consecutively form aggregates inside the cell [70]. In analogy with PD, where αSN levels are also found to be reduced in the cerebrospinal fluid, the protein may be trapped in αSN aggregates and thus no longer contribute to αSN serum levels [71]. While the histological findings are supportive in this regard, we did not find significant differences in αSN concentrations in muscle homogenates, which could be due to the possible confounding effect of haemolysis [51, 72, 73]. In this regard, significantly elevated Hb concentrations were observed in all examined tissue homogenate samples, and the observed αSN concentrations in tissue lysates most likely did not reflect the actual concentration in muscle cells. Furthermore, Kumar et al. reported a variable ability of different anti–αSN antibodies to detect monomeric, oligomeric or fibrillar αSN [74]. It appears reasonable that the aggregated αSN observed in histology may not be fully detected by the antibody used in the ELISA.

This study has some limitations. While the overall size of the cohort studied seems adequate given the rarity of the disease, some subgroups are comparatively small. Because of the partly retrospective study design, there are incomplete clinical and paraclinical data, which may confound correlation analyses. Therefore, prospective multicentre longitudinal studies in larger cohorts are mandatory. Finally, the classification of the individual cell types (e.g., atrophic and regenerating) was achieved based on their morphology in HE staining. However, co‐staining with cell type–specific markers next to αSN would be the gold standard to identify the presence of αSN reactivity in these cells. Consequently, the method used in this study may have misclassified a small proportion of αSN‐positive fibres. However, the main findings are likely to be valid regardless, especially as comparative results are observed when all fibres are included.

In conclusion, this study provides evidence for the use of αSN immunostaining in the diagnosis of IBM. αSN immunoreactivity may complement the established set of histological markers already used in clinical practice for the diagnosis of IBM. Furthermore, this study suggests reduced serum αSN concentrations as a candidate monitoring biomarker for IBM and encourages future (longitudinal) studies to elucidate the potential pathological role of αSN in muscle diseases and its applicability as a biomarker.

## Author Contributions

T.M., L.S., A.M. and L.F.: conception and design of the study, acquisition and analysis of data and drafting manuscript and figures. I.S., G.S.‐D., A.S., K.‐S.D., T.K., A.K., K.K., L.B. and M.O.: acquisition and analysis of data. All authors take full responsibilty for the critical revision of the manuscript.

## Ethics Statement

This study was performed in accordance with the ethical standards laid down in the 1964 Declaration of Helsinki and its later amendments. Approval was granted by the ethical committee of Martin Luther University of Halle‐Wittenberg (vote no. 2021‐101).

## Consent

Informed consent was obtained from all included patients.

## Conflicts of Interest

The authors declare no conflicts of interest.

## Supporting information


**Figure S1** Representative examples of quantification analysis of αSN‐stained samples. Quantification analysis was performed using ImageJ software. Every biopsy sample was analysed vertically and, if necessary, horizontally in a digital high‐power field until approximately 350 fibres were marked. Every fibre was assigned to one or more of the categories listed in the Method section, if applicable. Scale bars: 1000 μm.
**Figure S2** Serial section histology of fibres showing a subsarcolemmal enhancement of αSN reactivity. No clear association to lipofuscin, glycogen or accumulating mitochondria was observed. Abbreviations: αSN, α‐synuclein stain; AP, acid phosphatase stain; HE, haematoxylin eosin stain; NADH, nicotinamide adenine dinucleotide stain; PAS, periodic acid–Schiff stain; SDH, succinic dehydrogenase stain; and TR, Gömöri trichrome stain. Scale bar: 50 μm.
**Figure S3** Representative examples of αSN immunoreactivity in IBM and IMNM samples, illustrating no substantial difference in the staining pattern. Abbreviations: αSN, α‐synuclein stain; HE, haematoxylin eosin stain; IBM, inclusion body myositis; IMNM, immune‐mediated necrotising myopathy. Scale bars: ×20 50 μm and ×40 20 μm.
**Figure S4** Correlation analysis with histological and clinical parameters in IBM. A significant correlation of αSN reactivity with other histological markers of degeneration was observed, while there was no correlation with clinical parameters of disease severity. Abbreviations: αSN, α‐synuclein; ATR, atrophic fibres; CK, creatine kinase; FSLR, fibres with sarcolemmal enhancement of reactivity; FSPR, fibres with sarcoplasmic reactivity; IBM, inclusion body myositis; modified MRC‐SS, modified Medical Research Council sum score; NEC, necrotic fibres; VAC, vacuolar fibres; REG, regenerating fibres; *r,* reactive; and *t,* total.
**Figure S5** Analysis of αSN and Hb relationships in different samples and adjustment methods. (a) No clear correlation between Hb and αSN was seen graphically, while a slight correlation was suggested by statistical analysis in the diseased cohort (*r* = 0.525). In healthy controls, no correlation was observed. (b) In muscle tissue homogenates, Hb concentration was higher compared to serum samples. (c) In homogenates, an exponential relationship between αSN and Hb concentrations was observed (left). Semi‐log transformation of the relationship resulted in a linearization (right), which allowed for the calculation of a log‐αSN/Hb ratio to adjust for haemolysis. (d) After adjustment, no difference in log‐αSN/Hb ratio between the groups was observed. Abbreviations: CTR, control samples; αSN, α‐synuclein; Hb, haemoglobin; HER, hereditary myopathies; IBM, inclusion body myositis; and IIM, idiopathic inflammatory myopathies.
**Table S1** Antibody and kits used in this study.
**Table S2** Results of simple and multiple linear regression analyses in serum control samples.

## Data Availability

All data used for this study are available on reasonable request by contacting the authors.
