# Supplementary material for: Alpha‐Synuclein as a Potential Biomarker for Inclusion Body Myositis in Blood and Muscle
Source: Neuropathol Appl Neurobiol. 2025 May 19;51(3):e70019. doi: 10.1111/nan.70019 (PMC12086613; doi:10.1111/nan.70019)
Supplement: Supplementary file 1 — Figure S1 Representative examples of quantification analysis of αSN‐stained samples. Quantification analysis was performed using ImageJ software. Every biopsy sample was analysed vertically and, if necessary, horizontally in a digital high‐power field until approximately 350 fibres were marked. Every fibre was assigned to one or more of the categories listed in the Method section, if applicable. Scale bars: 1000 μm. Figure S2 Serial section histology of fibres showing a subsarcolemmal enhancement of αSN reactivity. No clear association to lipofuscin, glycogen or accumulating mitochondria was observed. Abbreviations: αSN, α‐synuclein stain; AP, acid phosphatase stain; HE, haematoxylin eosin stain; NADH, nicotinamide adenine dinucleotide stain; PAS, periodic acid–Schiff stain; SDH, succinic dehydrogenase stain; and TR, Gömöri trichrome stain. Scale bar: 50 μm. Figure S3 Representative examples of αSN immunoreactivity in IBM and IMNM samples, illustrating no substantial difference in the staining pattern. Abbreviations: αSN, α‐synuclein stain; HE, haematoxylin eosin stain; IBM, inclusion body myositis; IMNM, immune‐mediated necrotising myopathy. Scale bars: ×20 50 μm and ×40 20 μm. Figure S4 Correlation analysis with histological and clinical parameters in IBM. A significant correlation of αSN reactivity with other histological markers of degeneration was observed, while there was no correlation with clinical parameters of disease severity. Abbreviations: αSN, α‐synuclein; ATR, atrophic fibres; CK, creatine kinase; FSLR, fibres with sarcolemmal enhancement of reactivity; FSPR, fibres with sarcoplasmic reactivity; IBM, inclusion body myositis; modified MRC‐SS, modified Medical Research Council sum score; NEC, necrotic fibres; VAC, vacuolar fibres; REG, regenerating fibres; r, reactive; and t, total. Figure S5 Analysis of αSN and Hb relationships in different samples and adjustment methods. (a) No clear correlation between Hb and αSN was seen graphically, while a slight [file NAN-51-e70019-s001.pdf]

Electronic supplementary material

Article name: Alpha-synuclein as a potential biomarker for inclusion body myositis in blood and muscle

Authors: Tobias Mayer, Leila Scholle, Laura Foerster, Ilka Schneider, Gisela Stoltenburg-Didinger, Karl-Stefan Delank, Thomas Kendzierski, Anna Koelsch, Kathleen Kleeberg, Torsten Kraya, Lorenzo Barba, Steffen Naegel, Anne Schänzer, Markus Otto, Alexander Mensch

Submitting and corresponding author:
Alexander Mensch, MD
Department of Neurology, University Medicine Halle
Ernst-Grube-Str. 40, 06120 Halle (Saale), Germany
Tel.: +49 345 557 2856, Fax: +49 345 557 2860
E-Mail: alexander.mensch@medizin.uni-halle.de

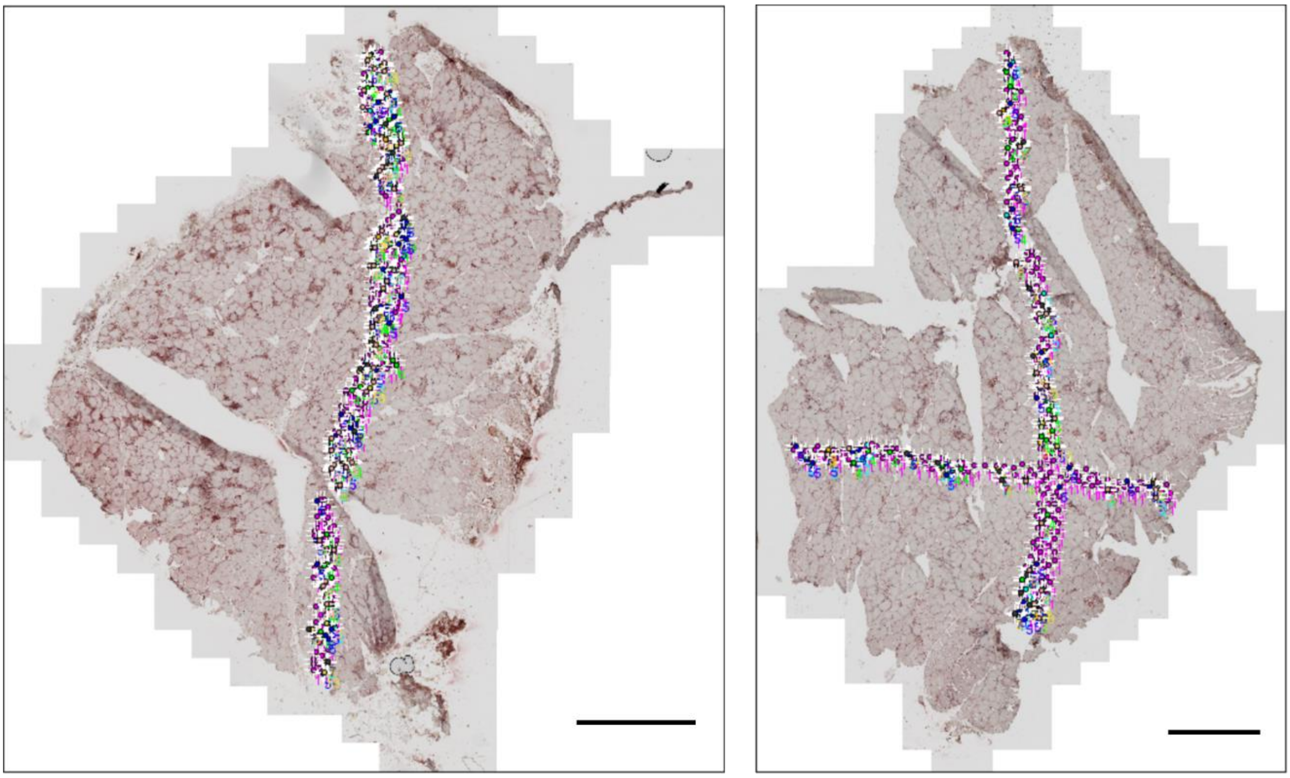

Suppl. Figure 1 Representative examples of quantification analysis of αSN-stained samples. Quantification analysis was performed using ImageJ software. Every biopsy sample was analysed vertically and, if necessary, horizontally in a digital high-power field until approximately 350 fibres were marked. Every fibre was assigned to one or more of the categories listed in the method section, if applicable. Scale bars: 1000 µm

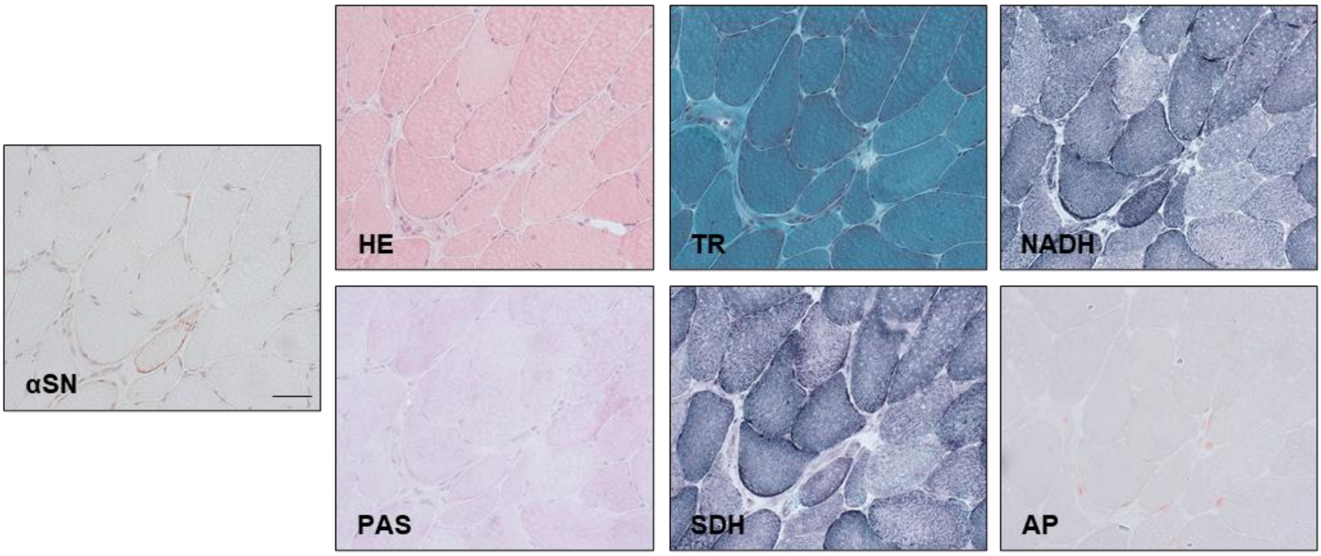

Suppl. Figure 2 Serial section histology of fibres showing a subsarcolemmal enhancement of αSN reactivity. No clear association to lipofuscin, glycogen or accumulating mitochondria was observed. *Abbreviations: αSN* – α-synuclein stain, *HE* – haematoxylin eosin stain, *TR* – Gömöri trichrome stain, *NADH* – nicotinamide adenine dinucleotide stain, *PAS* – periodic acid-Schiff stain, *SDH* – succinic dehydrogenase stain, *AP* – acid phosphatase stain; scale bar: 50 µm

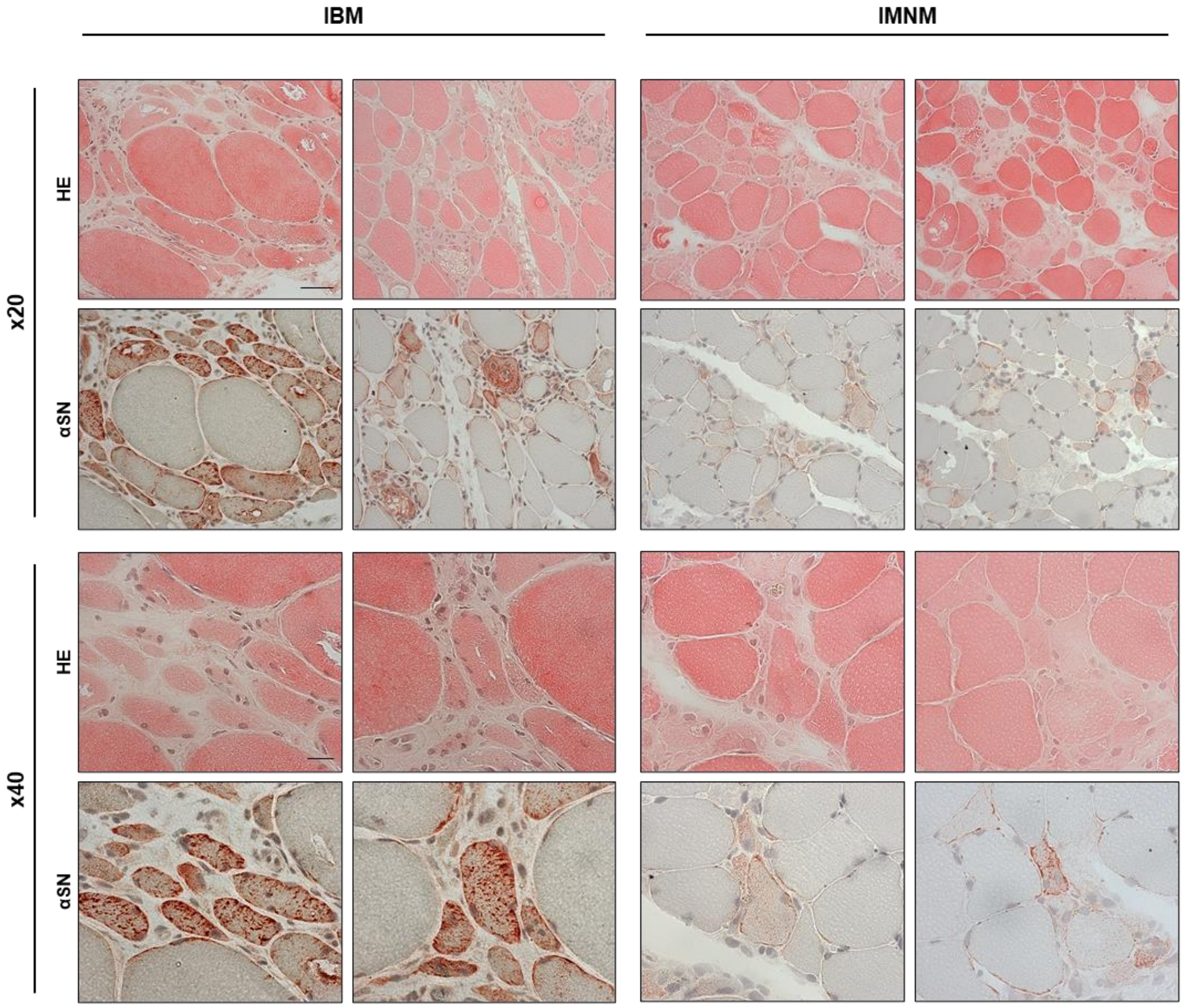

Suppl. Figure 3 Representative examples of αSN immunoreactivity in IBM and IMNM samples, illustrating no substantial difference in the staining pattern. *Abbreviations: IBM* – inclusion body myositis, *IMNM* – immune-mediated necrotizing myopathy, *αSN* – α-synuclein stain, *HE* – haematoxylin eosin stain; scale bars: **x20** 50 µm, **x40** 20 µm

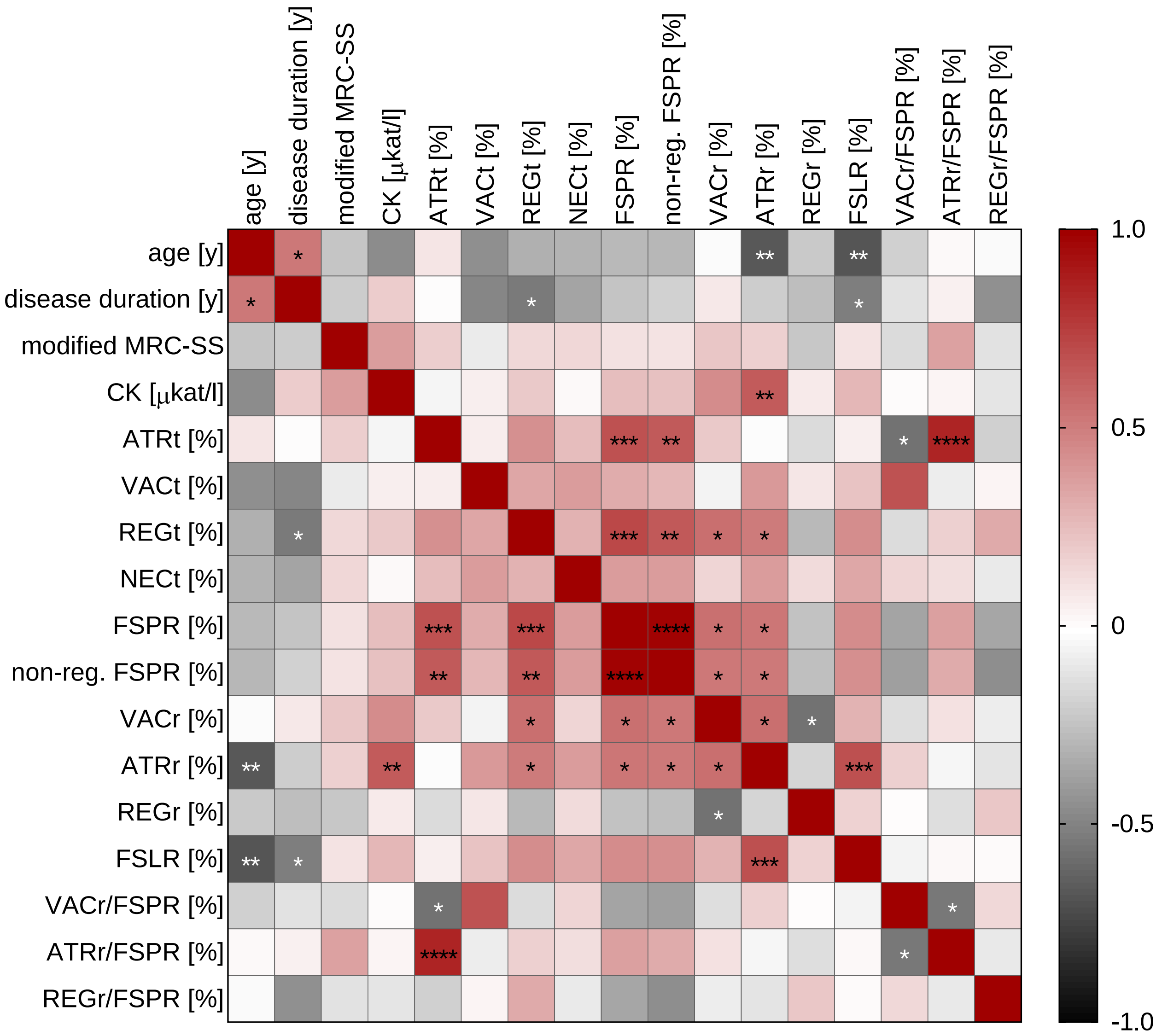

Suppl. Figure 4 Correlation analysis with histological and clinical parameters in IBM. A significant correlation of αSN reactivity with other histological markers of degeneration was observed, while there was no correlation with clinical parameters of disease severity. *Abbreviations: IBM* – inclusion body myositis, *αSN* – α-synuclein, *modified MRC-SS* – modified Medical-Research-Council sum-score, *CK* – creatine kinase, *ATR* – atrophic fibres, *VAC* – vacuolar fibres, *REG* – regenerating fibres, *NEC* – necrotic fibres, *FSPR* – fibres with sarcoplasmic reactivity, *FSLR* – fibres with sarcolemmal enhancement of reactivity, *t* – total, *r* – reactive

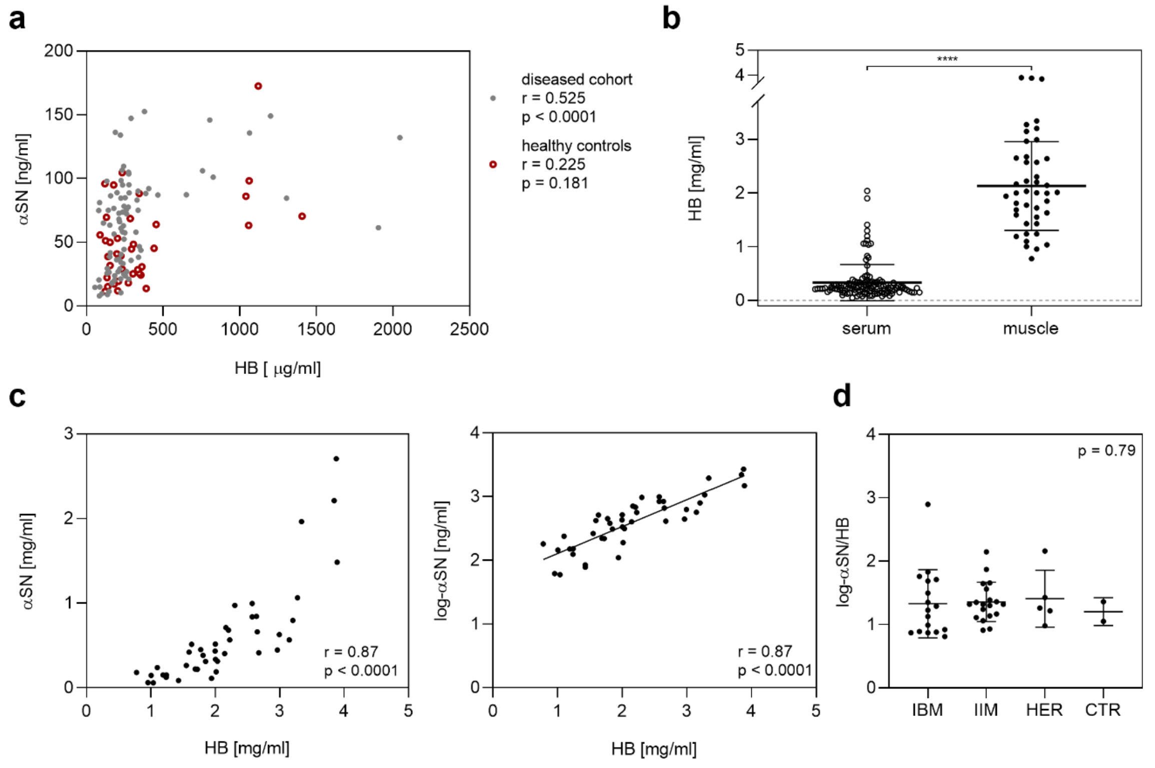

Suppl. Figure 5 Analysis of αSN and HB relationships in different samples and adjustment methods **a** No clear correlation between HB and αSN was seen graphically, while a slight correlation was suggested by statistical analysis in the diseased cohort (r = 0.525). In healthy controls, no correlation was observed. **b** In muscle tissue homogenates, HB concentration was higher compared to serum samples. **c** In homogenates, an exponential relationship between αSN and HB concentration was observed (left). Semi-log transformation of the relationship resulted in a linearization (right) which allowed for the calculation of a log-αSN/HB ratio to adjust for haemolysis. **d** After adjustment, no difference in log-αSN/HB ratio between the groups was observed. *Abbreviations: IBM* – inclusion body myositis, *IIM* – idiopathic inflammatory myopathies, *HER* – hereditary myopathies, *CTR* – control samples, *αSN* – α-synuclein, *HB* – haemoglobin

Suppl. Table 1 Antibody and kits used in this study

Antibody	Manufacturer	Catalogue Number	Clone	Dilution	Incubation conditions
α-synuclein	Santa Cruz Biotechnology, Dallas, Texas USA	sc-58480	LB 509	1:100	1 hour, room temperature

Kit	Manufacturer	Catalogue Number
AEC Substrate Kit	ZYTOMED Systems, Berlin, Germany	ZUC042-500
ZytoChem-Plus HRP Polymer-Kit	ZYTOMED Systems, Berlin, Germany	POLHRP-100
Human Alpha-synuclein SimpleStep ELISA Kit	Abcam, Cambridge, UK	ab260052
Alpha-Synuclein-ELISA	Euroimmun, Lübeck, Germany	EQ 6545-9601-L
Hemoglobin Human ELISA Kit	Abcam, Cambridge, UK	ab157707
Pierce™ BCA Protein Assay Kit	Thermo Fisher Scientific, Waltham, Massachusetts, USA	A55864

Suppl. Table 2 Results of simple and multiple linear regression analyses in serum control samples

variables	αSN [ng/ml]								
	simple linear regression analyses				multiple linear regression analysis				
	β	95% CI	R^2	p-value	β	95% CI	|t|	R^2	p-value
age [y]	0.8002	0.2151 - 1.385	0.1805	0.0088	0.5643	-0.07046 - 1.199	1.807	0.2459	0.0797
CK [µkat/l]	4.253	1.273 - 7.233	0.1935	0.0064	5.186	-0.9515 - 11.32	17.171		0.095

Abbreviations: αSN – α-synuclein, *y* – years, *CK* – creatine kinase, *95% CI* – 95% confidence interval
